# Control of *Meloidogyne incognita* in sweetpotato with fluensulfone

**DOI:** 10.21307/jofnem-2019-018

**Published:** 2019-04-26

**Authors:** Antoon Ploeg, Scott Stoddard, J. Ole Becker

**Affiliations:** 1 Department of Nematology, University of California Riverside, 3401 Watkins drive, Riverside, CA, 92521; 2 UCCE Merced and Madera Counties, 2145 Wardrobe Ave, Merced, CA, 95341

**Keywords:** Fluensulfone, *Ipomea batatas*, *Meloidogyne incognita*, Management, Nimitz, Sweetpotato

## Abstract

In California, sweetpotato is mostly grown on light sandy soils in Merced County. Root-knot nematodes (*Meloidogyne* spp.) can reduce sweetpotato yields and quality. Fluensulfone is the active ingredient of the new non-fumigant nematicide Nimitz. Unlike fumigant nematicides, toxicity toward non-target organisms is low, and it does not emit volatile organic compounds which negatively impact air quality. In two field trials, the effect of fluensulfone on *M. incognita* levels, and on the yield and quality of sweetpotato was determined. Fluensulfone was applied as a pre-plant soil incorporated drench or as a drench followed by post-plant sprays. Fluensulfone treatments more than doubled the marketable yields over an untreated control and a metam-sodium treatment in both trials. It strongly reduced nematode symptoms on the harvested roots and nematode infestation of these roots. The lowest rate of fluensulfone was as effective as the higher rates, and post-plant sprays following a pre-plant soil incorporated drench did not result in any additional benefits. Fluensulfone did not reduce soil nematode levels at harvest. It was concluded that a pre-plant incorporated fluensulfone drench at a rate of 1.96 kg/ha could provide a viable alternative for currently used nematicides to mitigate root-knot nematode damage in sweetpotato.

Sweetpotato (*Ipomea batatas*) production in California was approximately 295 million kg annually during 2010 to 2015 grown on approximately 7,300 ha. California production is second only to North Carolina, and the crop in California is valued at $150 million, which is about 20% of the total US value. Close to 90% of the production in California is concentrated on the sandy soils of Merced County in the San Joaquin Valley (USDA/NASS). Planting material is typically produced in plastic tunnels (hotbeds) by planting sweetpotato roots from the previous year. After sprouting, the stems are cut, and these stem cuttings or ‘slips’ which do not have any roots, are used as planting material in April to May in the production fields (about 37,000 slips per hectare) (Stoddard et al., 2013). In California production fields, the crop is usually grown in double rows on 203 cm-wide (center to center) beds, and irrigation is through surface drip tubing on the center of the bed (Stoddard et al., 2013).

Root-knot nematodes (RKN: *Meloidogyne* spp.) are economically the most damaging nematodes in sweetpotato both on a worldwide scale as well as in California (Overstreet, 2009). Crop loss estimates of 10% due to RKN were reported in California (Koenning et al., 1999). Unlike many other vegetable crops, most sweetpotato cultivars are particularly sensitive to RKN damage because symptoms develop directly on the harvested product. Symptoms of RKN on the harvested storage roots depend on the sweetpotato cultivar but generally include blistering or bumpiness of the storage root surface (Overstreet, 2009). Some cultivars may exhibit cracking of the storage roots. Lawrence et al. (1986) suggested that RKN predispose the roots to cracking when soil moisture levels fluctuate during the development of the storage roots, rather than directly causing this symptom. Generally, RKN females and egg masses are easily found embedded in the storage roots just below the surface and may be associated with pinpoint necrotic spots (Lawrence et al., 1986). Apart from a reduction in quality, a general reduction in yield (kg/ha) is also common (Roberts and Scheuerman, 1984; Overstreet, 2009). Economic damage thresholds for the RKN species *M. incognita* depend on the cultivar and environmental factors, but Ferris (1978) reported a threshold level of 5 s-stage juveniles (J2) per 1 kg soil for a sandy soil. Lawrence et al. (1986) found a damage threshold of 10 J2 per 500 cm^3^ soil for cracking of storage roots. Overstreet (2009) and Stoddard et al. (2013) also hint at very low threshold levels.

Some cultivars (e.g. Covington, Murasaki) have good RKN resistance, but under high soil temperatures, even resistant cultivars can still result in a large RKN population increase during one crop cycle (Roberts and Scheuerman, 1984). Furthermore, although storage root quality of resistant cultivars was not affected by RKN, yield losses resulting from RKN were still considerable, and additional management strategies are needed in RKN infested fields, even when growing RKN-resistant cultivars (Roberts and Scheuerman, 1984).

Typically soil fumigants are used to control RKN both in nursery hotbeds and in production fields. According to 2015 data (CA-DPR), sweetpotato was among the five crops in California with the highest use of the fumigant 1,3-dichloropropene (2,999 ha). Other fumigants used in sweetpotato in California are metam-potassium (809 ha) and metam-sodium (33 ha). As they are potential environmental and health hazards, they are limited by regulatory restrictions related to the emission of volatile organic compounds (VOC) and their toxicity. Until recently, effective, environmentally acceptable, and economically viable alternatives were not available, and this has been an important factor in the continued use of soil fumigants (Noling and Becker, 1994; Becker, 2014). Fluensulfone (tradename: Nimitz, ADAMA Agricultural Solutions Ltd., Raleigh, NC) is a non-fumigant nematicide that is registered for use in fruiting vegetable crops in California. It has a ‘caution’ label and no re-entry interval (0 hr REI) after application. The product is applied pre-plant, either by chemigation through the drip tubing, or by soil incorporation at rates between 4.1 and 5.8 liter/ha (www.adama.com). Studies on RKN control by fluensulfone in tomato, carrot, tobacco, and cucumber showed promising results (Csinos et al., 2010; Becker et al., 2013; Dickson and Mendes, 2013; Ploeg et al., 2013; Morris et al., 2015, 2016). Although Dickson and Mendes (2013) mention a yield increase in sweetpotato after a fluensulfone application, they do not provide further information.

The goal of this two-year field study was to evaluate the effectiveness of fluensulfone in comparison to an untreated control and to metam-sodium in sweetpotato grown on an uniformly *M. incognita*-infested site.

## Materials and methods

The trials were located on a field with sandy-loam soil (70% sand, 18% silt, 12% clay, 0.1% organic matter, pH 7.3) at the University of California South Coast Research and Extension Center, Irvine, CA. The field had been inoculated five years previously with an egg suspension of a *M. incognita* race 3 population, originally isolated from cotton in the San Joaquin Valley, CA, by injecting the egg suspension through buried drip tubing (Becker et al., 1989). The *M. incognita*-susceptible crops melon (*Cucumis melo* ‘Durango’), carrot (*Daucus carota* ‘Imperator 58’), tomato (*Solanum lycopersicum* ‘Halley 3155’), and bean (*Phaseolus vulgaris* ‘Blue Lake 274’) were grown in sequence during the spring/summer for four years to increase and maintain an evenly distributed *M. incognita* infestation level before the sweetpotato trial was initiated. Wheat (*Triticum aestivum*) ‘Yecora Rojo’ was grown during the winter each year.

The trials were conducted in 2016 and 2017 on different, but nearby areas of the field. In both years, 152 cm wide (center to center) beds were prepared in May and plots were laid out. Individual plots were 6.1 m long sections of bed, separated along the beds by a 91 cm border section. The experiment was designed according to a completely randomized block design with five replicates and four treatments. In both years treatments included an untreated control, a Vapam (a.i. metam-sodium) treatment at 701 liter/ha (294 liter a.i./ha), and two fluensulfone treatments. In 2016, the fluensulfone treatments were (i) Nimitz at 7 liter/ha (3.36 kg a.i./ha, pre-plant incorporated) and (ii) Nimitz at 7 liter/ha (3.36 kg a.i./ha, pre-plant incorporated) followed by two post-plant spray applications of 3.5 liter/ha (1.68 kg a.i./ha) at 26 and 58 d after planting. In 2017, fluensulfone treatments were (i) Nimitz at 5.8 liter/ha (2.8 kg a.i./ha) and (ii) Nimitz at 4.1 liter/ha (1.96 kg a.i./ha) both pre-plant incorporated. Vapam was applied 21 and 26 d before planting in 2016 and 2017, respectively. Pre-plant Nimitz applications were 2 and 7 d before planting in 2016 and 2017, respectively. Amounts applied per plot were based on the bed surface area of each plot (5.88 m^2^). All plots were pre-irrigated for 1 hr with overhead sprinklers the day prior to any pre-plant application to achieve adequate soil moisture. For each plot, Vapam and pre-plant Nimitz were suspended in 7.6 liter of water and watered evenly over the plot surface with a watering can. An additional 45.4 liter of water was applied over each plot, and the plots were tilled with a rototiller to a depth of 10 to 13 cm. Post-plant Nimitz applications were applied in 7.6 liter of water with a backpack sprayer over the crop foliage.

For RKN analysis, a composite sample consisting of six cores of soil (1.5 cm diameter, 5–30 cm depth) was collected from each plot just before applying Vapam (initial population: Pi) and just before harvest (final population: Pf). Nematodes were extracted from 100 g soil subsamples in a modified Baerman-funnel technique (Rodriguez-Kabana and Pope, 1981), and RKN J2 were counted at ×40 magnification.

Rootless slips of the RKN-susceptible cultivars O’Henry and Beauregard were planted on June 10, 2016 and May 18, 2017, respectively. The slips were planted in pre-wetted beds at 41 cm within-row spacing, with two rows per bed, resulting in 30 slips per plot. At planting, approximately 0.5 liter water was added to each cutting, and irrigation was through drip tubing (drip emitters 2 liter/hr, 30.5 cm spacing) on top and in the center of the beds. Fertilization was according to standard practices, applied pre-plant incorporated and post-plant through the drip tubing. Weeds were removed by hand, and no fungicides or insecticides were required. In total, 20 and 50 d after planting, the general vigor of each plot was visually examined and indexed (1–10 scale). Plots were harvested mechanically on October 9, 2016 and September 22, 2017. For each plot, total yields (weight and number of roots) were determined. In total, 20 roots were randomly collected from each plot, and assigned to one of three categories: marketable, non-marketable because of RKN damage, and non-marketable because of defects not related to RKN. The weight of these roots in each category was determined. In addition, 10 randomly selected roots from each plot were taken to the laboratory and cut in half cross-wise. One half was discarded. The 10 remaining half roots were weighed and then peeled with a potato peeler. Nematode eggs were extracted from both the peels and the peeled roots by shaking for 3 min in a 0.5% NaOCl solution (Hussey and Barker, 1973) and collected by washing over two stacked 25 μm pore-size sieves. The eggs were counted at ×40 magnification.

### Statistical analysis

Treatment effects on nematode counts, crop vigor, sweetpotato yield, and sweetpotato quality were analyzed using an analysis of variance (ANOVA) procedure, and means were compared using Fisher’s protected least significant difference (LSD) test (*P* ⩽ 0.05) using SAS statistical software (SAS Institute, Cary, NC, USA). Percentage data were transformed by arcsin (√x) before statistical analysis, nematode counts were transformed by *x* ^1^ = log_10_ (*x* + 1) before statistical analysis.

## Results

General growing conditions for the trial were excellent in both years, and nearly 100% of planted slips survived. In both trial years, crop vigor was not affected by the treatments (Table 1). In 2016, effects of the two fluensulfone treatments on sweetpotato yields (kg) were highly significant. Both fluensulfone treatments more than doubled the overall yield relative to the untreated control (Table 2). In 2017, the fluensulfone treatments yielded about 9 kg/plot more than the untreated controls, but these differences were not significant. In both years, the fluensulfone treatments dramatically increased the marketable yield compared to the untreated control. The metam-sodium treatment failed to improve sweetpotato yields (quantity, quality) and was not significantly better than the untreated control. When examining the yields as percentages from the total yield, the same general effects exist (Table 3). Compared to the untreated control, the percentage of harvested roots culled because of obvious RKN symptoms (bumpiness, cracking) was reduced by the fluensulfone treatments in both years. In 2016, the percentage of roots culled because of other reasons (insect damage, too small, misshapen) was significantly higher in both fluensulfone treatments, but this was not the case in 2017. Metam-sodium treatments did not significantly affect the relative tuber yields in the three different quality classes (marketable, cull RKN, cull other) compared to the untreated control in either year.

**Table 1:** Average (*n* = 5) vigor of sweetpotato cultivars O’Henry (2016) and Beauregard (2017) in four treatments 20 and 50 d post-plant. Field located at SCREC, Irvine, CA^1^. Vigor rating from 1 to 10 (very poor − excellent) ± standard error.

	Vigor rating (days after planting)	
Treatment	20	50
*2016*		
1. Untreated Control	7.4 ± 0.89	7.2 ± 0.84
2. Metam-sodium (294 liter/ha)	8.0 ± 0.71	7.8 ± 0.45
3. Fluensulfone pre-plant (3.36 kg/ha)	7.6 ± 0.89	7.6 ± 0.55
4. Fluensulfone pre-plant (3.36 kg/ha) and 2× post (1.68 kg/ha + 1.68 kg/ha)	7.8 ± 0.45	7.6 ± 0.55
treatment *P-value*	0.62	0.56
*2017*		
1. Untreated Control	4.8 ± 0.49	6.0 ± 0.32
2. Metam-sodium (294 liter/ha)	6.0 ± 0.89	6.2 ± 0.37
3. Fluensulfone pre-plant (1.96 kg/ha)	7.2 ± 0.66	7.2 ± 0.37
4. Fluensulfone pre-plant (2.8 kg/ha)	6.4 ± 0.81	6.4 ± 0.40
Treatment *P-value*	0.19	0.20

**Notes:** ^a^Plot size: 6.1 m long section of 152-cm wide beds. Two lines of sweetpotato planted per bed.

**Table 2:** Average yield (*n* = 5 ± standard error) of harvested sweetpotato after four treatments assigned to three categories, market (marketable size and quality), cull RKN (culled because of root-knot nematode damage), and cull other (culled because of non-nematode causes). Field trials were conducted during 2016 (cultivar O’Henry) and 2017 (cultivar Beauregard) at SCREC, Irvine, CA^1^.

	Sweetpotato Yield (kg/plot^a^)							
Treatment	Total		Market		Cull RKN		Cull other	
*2016*								
1. Untreated Control	14.9 ± 1.5	b^b^	0.8 ± 0.4	b	10.5 ± 1.5	a	3.6 ± 1.2	b
2. Metam-sodium (294 liter/ha)	19.7 ± 5.0	b	0.9 ± 0.3	b	11.7 ± 1.5	a	7.0 ± 3.5	b
3. Fluensulfone pre-plant (3.36 kg/ha)	29.6 ± 3.5	a	8.2 ± 0.2	a	4.6 ± 1.3	b	16.8 ± 3.0	a
4. Fluensulfone pre-plant (3.36 kg/ha) and 2× post (1.68 kg/ha + 1.68 kg/ha)	29.8 ± 3.0	a	10.1 ± 0.4	a	3.6 ± 0.7	b	16.1 ± 2.6	a
Treatment *P-*value	0.01		0.0001		0.0003		0.006	
*2017*								
1. Untreated Control	24.8 ± 2.7	a	6.7 ± 1.9	b	15.0 ± 3.9	a	3.1 ± 0.6	a
2. Metam-sodium (294 liter/ha)	27.7 ± 2.8	a	9.9 ± 1.0	b	12.5 ± 2.7	a	5.3 ± 1.2	a
3. Fluensulfone pre-plant (1.96 kg/ha)	34.0 ± 2.4	a	18.4 ± 2.6	a	12.0 ± 2.6	a	3.5 ± 1.4	a
4. Fluensulfone pre-plant (2.8 kg/ha)	33.0 ± 3.6	a	23.3 ± 3.4	a	7.1 ± 1.4	a	2.6 ± 0.8	a
Treatment *P-value*	0.13		0.002		0.32		0.30	

**Notes:** ^a^Plot size: 6.1 m long section of 152-cm wide beds. Two lines of sweetpotato planted per bed; ^b^different letters within the same column and within the same year represent significant differences at the 95% confidence level.

**Table 3:** Average percentage (*n* = 5 ± standard error) of harvested sweetpotato after four treatments assigned to three categories, market: marketable root size and quality, cull RKN: culled because of root-knot nematode damage, and cull other: culled because of non-nematode causes. Field trials during 2016 (cultivar ‘O’Henry’) and 2017 (cultivar ‘Beauregard’) at SCREC, Irvine, CA.

	Sweetpotato yield (%)					
Treatment	Market		Cull RKN		Cull other	
*2016*						
1. Untreated Control	6.6 ± 4.3	b^a^	70.3 ± 8.1	a	23.3 ± 6.0	b
2. Metam-sodium (294 liter/ha)	5.3 ± 2.3	b	66.3 ± 6.9	a	28.5 ± 7.4	b
3. Fluensulfone pre-plant (3.36 kg/ha)	28.3 ± 5.3	a	16.4 ± 5.5	b	55.4 ± 3.8	a
4. Fluensulfone pre-plant (3.36 kg/ha) and 2× post (1.68 kg/ha + 1.68 kg/ha)	35.1 ± 2.9	a	12.0 ± 2.1	b	53.0 ± 4.3	a
Treatment *P-value*	0.0001		0.0043		0.003	
*2017*						
1. Untreated Control	29 ± 8.0	c	58 ± 9.4	a	13 ± 3.0	a
2. Metam-sodium (294 liter/ha)	37 ± 4.0	bc	44 ± 7.0	ab	19 ± 4.3	a
3. Fluensulfone pre-plant (1.96 kg/ha)	54 ± 6.6	ab	35 ± 7.6	bc	11 ± 5.1	a
4. Fluensulfone pre-plant (2.8 kg/ha)	70 ± 3.2	a	21 ± 2.9	c	9 ± 2.9	a
Treatment *P-value*	0.005		0.02		0.27	

**Notes:** ^a^Different letters within the same column and within the same year represent significant differences at the 95% confidence level. Data were transformed by arcsin [√(*x*/100)] before statistical analysis, non-transformed data shown.

The average RKN J2 levels at the start of the trial were 15.8 and 47.5 J2 per 100 g soil in 2016 and 2017, respectively (Table 4). In both years, these pre-treatment nematode levels were not significantly different among the treatments. At harvest, nematode levels had increased about 13-fold in 2016 and 8-fold in 2017 and were not significantly different among the four treatments. In both years, the level of nematode infestation of the harvested sweetpotato roots however was significantly lowered by the fluensulfone treatments resulting in a reduction of the egg load of the roots by over 80% compared to the untreated control. The metam-sodium treatment did not result in a significant reduction in sweetpotato root infestation levels at harvest relative to the untreated control.

**Table 4:** Average root-knot nematode levels (*n* = 5 ± standard error) in soil and on harvested sweetpotato after four treatments. Field trials during 2016 (cultivar O’Henry) and 2017 (cultivar Beauregard) at SCREC, Irvine, CA.

	J2 per 100 g soil					
Treatment	Pre-plant (Pi)		Post-plant (Pf)		Eggs per g sweetpotato	
*2016*						
1. Untreated Control	23 ± 16	a^a^	198 ± 42	a	536 ± 38	a
2. Metam-sodium (294 liter/ha)	12 ± 5	a	300 ± 61	a	573 ± 133	a
3. Fluensulfone pre-plant (3.36 kg/ha)	14 ± 6		173 ± 51	a	79 ± 17	b
4. Fluensulfone pre-plant (3.36 kg/ha) and 2x post (1.68 kg/ha + 1.68 kg/ha)	14 ± 8	a	156 ± 33	a	98 ± 34	b
Treatment *P-value*	0.95		0.29		0.0001	
*2017*						
1. Untreated Control	21.2 ± 9.1	a	360 ± 107	a	304 ± 46	a
2. Metam-sodium (294 liter/ha)	25.0 ± 12.3	a	261 ± 52	a	228 ± 87	a
3. Fluensulfone pre-plant (1.96 kg/ha)	34.4 ± 14.4	a	396 ± 80	a	37 ± 16	b
4. Fluensulfone pre-plant (2.8 kg/ha)	49.0 ± 17.8	a	532 ± 132	a	21 ± 5	b
Treatment *P-value*	0.65		0.54		0.0005	

**Notes:** ^a^Different letters within the same column and within the same year represent significant differences at the 95% confidence level. Data were transformed by arcsin [√(x/100)] before statistical analysis, non-transformed data shown.

## Discussion

The earliest report of fluensulfone use against nematodes was from 2010 showing that the nematicide reduced root-galling and increased yield of tobacco grown in a *M. arenaria* infested site (Csinos et al., 2010). Since then, most studies on the use and efficacy of fluensulfone for nematode control have been done on fruiting vegetables. The registration of Nimitz (a.i. fluensulfone) in the USA was first obtained for these crops (Gine, 2016). Current registration also includes leafy vegetables, brassica vegetables, and strawberry. The efficacy of fluensulfone in root and tuber crops was also being tested such as in carrot (Ploeg et al., 2013; Westerdahl, 2014), potato (Norshie et al., 2016), and sweetpotato in this study. In these crops, the adverse impact of nematodes on the quality of the harvested product is often more significant than the impact on overall yield. In both years in our study, pre-plant nematode levels were at least 15 J2/100 g soil, which corresponds to approximately 120 J2/500 cm^3^. Because damage thresholds are estimated at only 10 J2/500 cm^3^, it is not surprising that in both years over 50% percent of roots were culled in the untreated control. Fluensulfone treatments increased the percentage of marketable sweetpotatoes in both years over the untreated control, but within the same year, fluensulfone treatments were not different. This indicates that post-plant spray applications did not provide an additional benefit after a pre-plant soil incorporated treatment (2016) and that a pre-plant soil incorporated rate of fluensulfone at 1.96 kg/ha was as effective as the 2.8 kg/ha rate (2017). Surprisingly, in 2016 the percentage of culled roots that did not show obvious signs of nematode damage (bumpy appearance, cracking) was significantly higher after the two fluensulfone treatments. This suggests that fluensulfone caused some other effect on the roots, e.g., increased the number of roots with insect damage, or resulted in more misshapen or small roots. An increase in insect (wireworm) damaged roots resulting from fluensulfone seems unlikely, but it could be that in 2016 Nimitz did reduce nematode symptoms of the roots, but at the same time had some phytotoxic effect resulting in more misshapen and smaller roots. This would explain the relatively higher percentage of ‘no-nematode’ culls in the fluensulfone treatments in 2016. In 2017, when lower rates were used, this effect did not occur. Phytotoxic effects of fluensulfone in vegetable crops have been reported when used at high rates, as a post-plant spray, or too close to planting time (Oka et al., 2012; Van Dyk et al., 2013; Morris et al., 2016). Stoddard (2010) observed early-season phytotoxic effects associated with MCW-2 (a.i. fluensulfone) treatments in a California sweetpotato field trial.

In both years, the RKN J2 soil populations at harvest were similar among the treatments. This was true also in previous field trials on carrot (Ploeg et al., 2013) and tomato (Becker et al., 2013), although others did find that fluensulfone resulted in significant reductions in RKN J2 populations at harvest in field or microplot trials with tobacco (Csinos et al., 2010), tomato (Morris et al., 2015), and lima bean (Jones et al., 2017). In our trials, the metam-sodium treatment did not differ from the untreated control. This may have been due to the relatively superficial incorporation of the product and the failure to provide an adequate seal post application. The positive effect of fluensulfone on the marketable root yield was reflected in its ability to strongly reduce nematode infestation of the harvested storage roots, even though soil RKN populations were not lowered. This suggests that nematode infestation of the storage roots was more effectively controlled than of the feeder roots and that the increase in nematode soil levels in the fluensulfone treatments was mostly the result of nematode multiplication in the feeder roots. Possibly, the developing young storage roots are most susceptible to RKN infestation, when the activity of fluensulfone is still high, and lose their susceptibility as they develop, while the feeder roots remain susceptible throughout the crop cycle. The observed outcome is similar to what Roberts and Scheuerman (1984) observed after growing nematode resistant sweetpotato cultivars: the storage roots remained virtually free of nematode symptoms while post-harvest soil RKN populations increased. Villordon et al. (2009) noted that storage root development in sweetpotato is largely determined in the first 17 d after transplanting.

These trials show that fluensulfone when applied as an incorporated soil drench at least 2 d before planting a nematode-susceptible sweetpotato cultivar in RKN (*M. incognita*) infested soil, significantly improves both yield and quality. Total root yields doubled, and a 10-fold increase in marketable yield occurred compared to the untreated control. The 1.96 kg/ha rate was as effective as the 2.8 kg/ha rate, and post-plant spray applications did not offer additional benefits. However, this lower rate will need to be evaluated in additional field trials before it can be recommended to sweetpotato producers. Fluensulfone failed to reduce soil RKN levels at harvest time but did reduce nematode infestation of sweetpotato roots by over 80%. We conclude that fluensulfone provides a viable new management option to growers in California for reducing RKN damage in sweetpotato that is both safe and effective.
